# Virtual Fracture Clinic: A Helping Hand for an Overburdened Traditional Fracture Clinic in a Busy Major Trauma Center

**DOI:** 10.7759/cureus.83901

**Published:** 2025-05-11

**Authors:** Masroor Ahmed, Mayank Kumar, Ahmad W Mohamed, Arman Amjad, Matija Krkovic

**Affiliations:** 1 Trauma and Orthopaedic Surgery, Addenbrooke's Hospital, Cambridge University Hospitals NHS Foundation Trust, Cambridge, GBR

**Keywords:** emergency department referrals, orthopaedic care, outpatient management, patient-initiated follow-up, virtual fracture clinic

## Abstract

Introduction

Virtual fracture clinics (VFCs) were established to improve the efficiency of orthopedic care by reducing unnecessary face-to-face consultations, decreasing waiting times, and providing timely specialist advice. This study aims to evaluate the utilization of the VFC at Addenbrooke’s Hospital, Cambridge University Hospital NHS Foundation Trust (Cambridge, GBR), over 12 months and assess its effectiveness in managing patients referred from emergency departments and minor injury units.

Methods

This retrospective study included all patients referred to the VFC between January 2023 and January 2024. Data were collected from hospital electronic records and analyzed using SPSS Statistics version 20.0 (IBM Corp., Armonk, NY, USA). Patients were assessed by a consultant orthopaedic surgeon based on referral details and imaging findings. Clinical outcomes were documented as face-to-face consultation, discharge with patient-initiated follow-up (PIFU), or referral to subspecialty clinics.

Results

A total of 5,034 patients were reviewed by the VFC. The most common injuries involved the wrist, hand, foot, ankle, shoulder, and knee. Of the total patients, 62% were discharged with PIFU, while 30.4% required face-to-face consultation. Among patients initially discharged with PIFU, 16.9% were subsequently rebooked into subspecialty clinics for further evaluation. Statistical analysis demonstrated a significant association between injury type and VFC outcome, indicating a moderate effect size.

Conclusions

The VFC model effectively managed a substantial proportion of patients referred from emergency departments and minor injury units, providing safe and efficient care while reducing the burden on traditional fracture clinics.

## Introduction

Virtual fracture clinics (VFCs) were first introduced at the Glasgow Royal Infirmary in 2011 [1]. Since then, they have gained popularity and been widely accepted by orthopaedic departments in the United Kingdom, Ireland, and worldwide. The COVID-19 pandemic significantly accelerated the adoption of VFCs to deliver health care [2,3]. These clinics have demonstrated safety in managing patients presenting to emergency departments who do not require immediate intervention or specialist treatment [4].

Despite the growing popularity of VFCs, concerns remain among healthcare administrators and providers regarding their safety, consistency, and patient satisfaction. However, recent evidence has shown that VFCs can safely and effectively manage injuries such as minimally displaced radial head fractures, Jones fractures, and stable Weber B fractures, with satisfactory outcomes [5-7]. The increasing number of referrals from emergency departments and minor injury units to VFCs has resulted in a substantial rise in workload, which has strained available resources. Approximately 75% of these referrals involve simple, non-displaced to minimally displaced stable fractures that are often unnecessarily immobilised, contrary to standardised management protocols [8]. Furthermore, most of these stable injuries do not require follow-up in a traditional face-to-face fracture clinic for clinical assessment [9]. This study aims to evaluate the utilisation of the VFC from January 2023 to January 2024, including the number of patients managed, the proportion requiring face-to-face consultation, and the number discharged following VFC review. Additionally, we sought to assess the impact of the VFC on reducing the workload of a busy orthopaedic outpatient clinic at a major trauma centre.

## Materials and methods

This retrospective study was conducted at Addenbrooke’s Hospital, Cambridge University Hospital NHS Foundation Trust (Cambridge, GBR), following approval from the Audit and Research Committee (clinical project approval no. 6195). Data were collected from the hospital’s electronic records department, including patients referred from the emergency department and minor injury units to the VFC from January 2023 and January 2024.

Patients referred to the VFC were assessed by a consultant orthopaedic surgeon based on referral details and imaging findings. The clinical decision was documented in patient records, and patients were contacted via telephone to discuss further management. Data collected included age, gender, the region of the body injured, and the VFC decision following consultation.

Statistical analysis

Descriptive statistics were used to summarise patient demographics, injury types, and clinical outcomes following VFC review. Categorical variables, including injury type and VFC outcome, were compared using the chi-square test of independence to evaluate the association between injury type and clinical outcomes. The strength of association was measured using the phi coefficient (Φ). A p-value of <0.05 was considered statistically significant. We used SPSS Statistics for Windows version 20.0 (IBM Corp., Armonk, NY, USA) to perform all statistical analyses.

## Results

**Figure 1:**
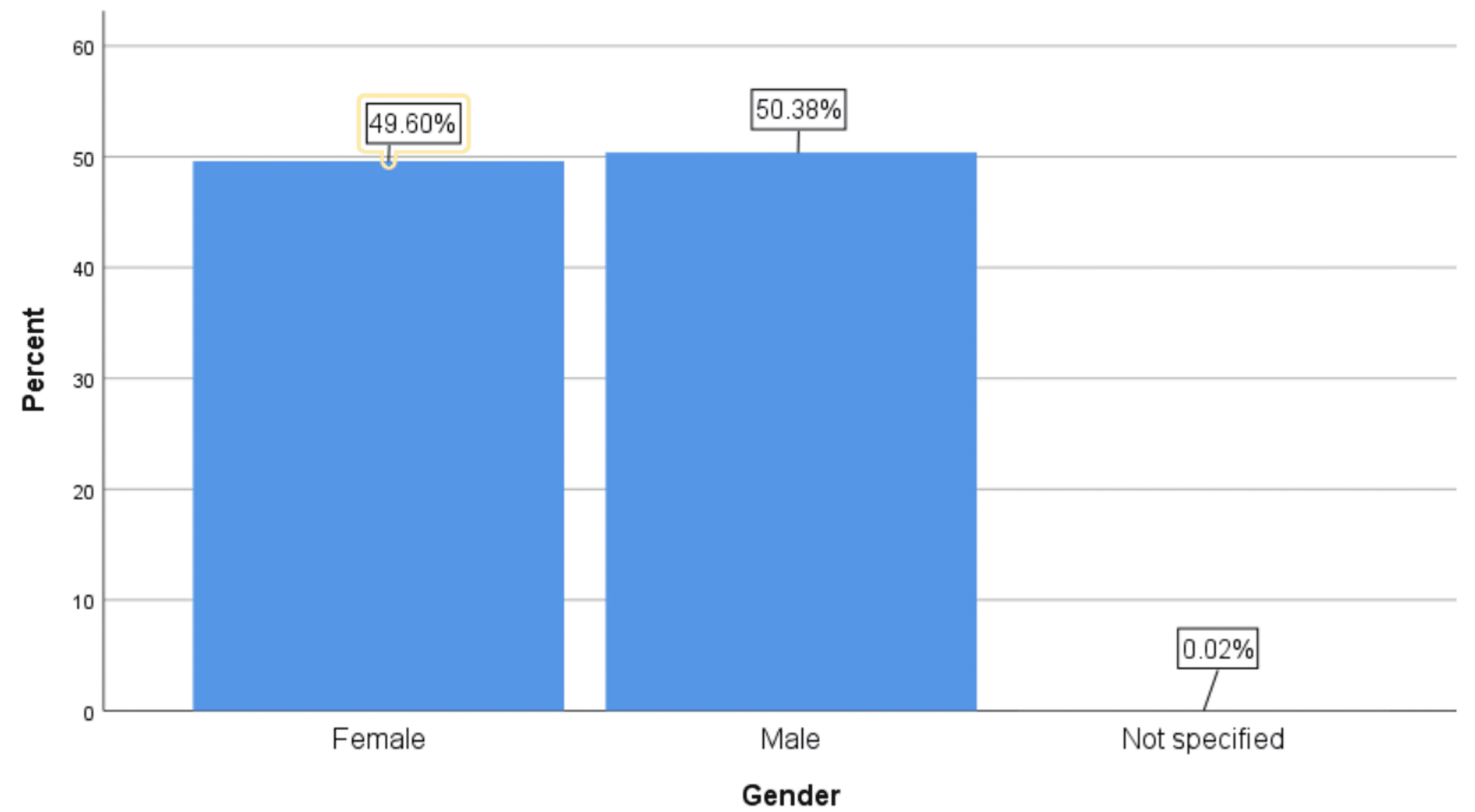
Patient gender distribution

**Figure 2:**
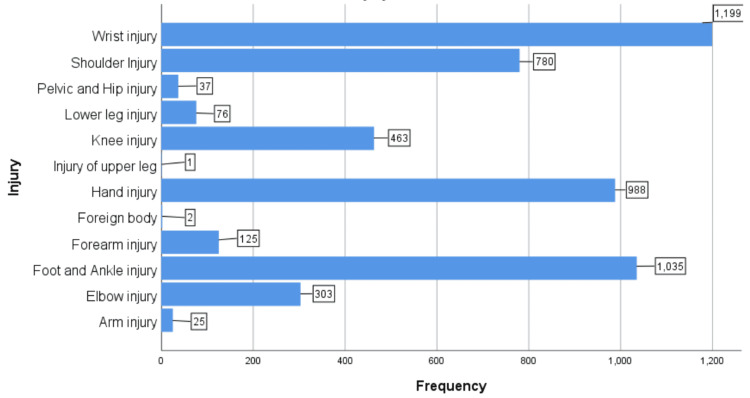
Distribution of injuries by affected body region

A total of 5,034 patients referred from the accident and emergency department were included in the study. Of these, 2,536 (50.4%) were men, 2,497 (49.6%) were women, and one patient (0.03%) did not have their gender specified (Figure 1). The mean age of referral was 36.7 years (range: 1 to 100 years). To simplify data presentation, injuries were categorised by the affected body region rather than specific fracture types. The most common referrals were for wrist injuries (1,199; 23.8%) and hand injuries (988; 19.6%), together accounting for 43.4% of all injuries. Foot and ankle injuries were observed in 1,035 (20.6%) patients, shoulder injuries in 780 (15.5%) patients, and knee injuries in 463 (9.2%) patients (Figure 2).

**Figure 3:**
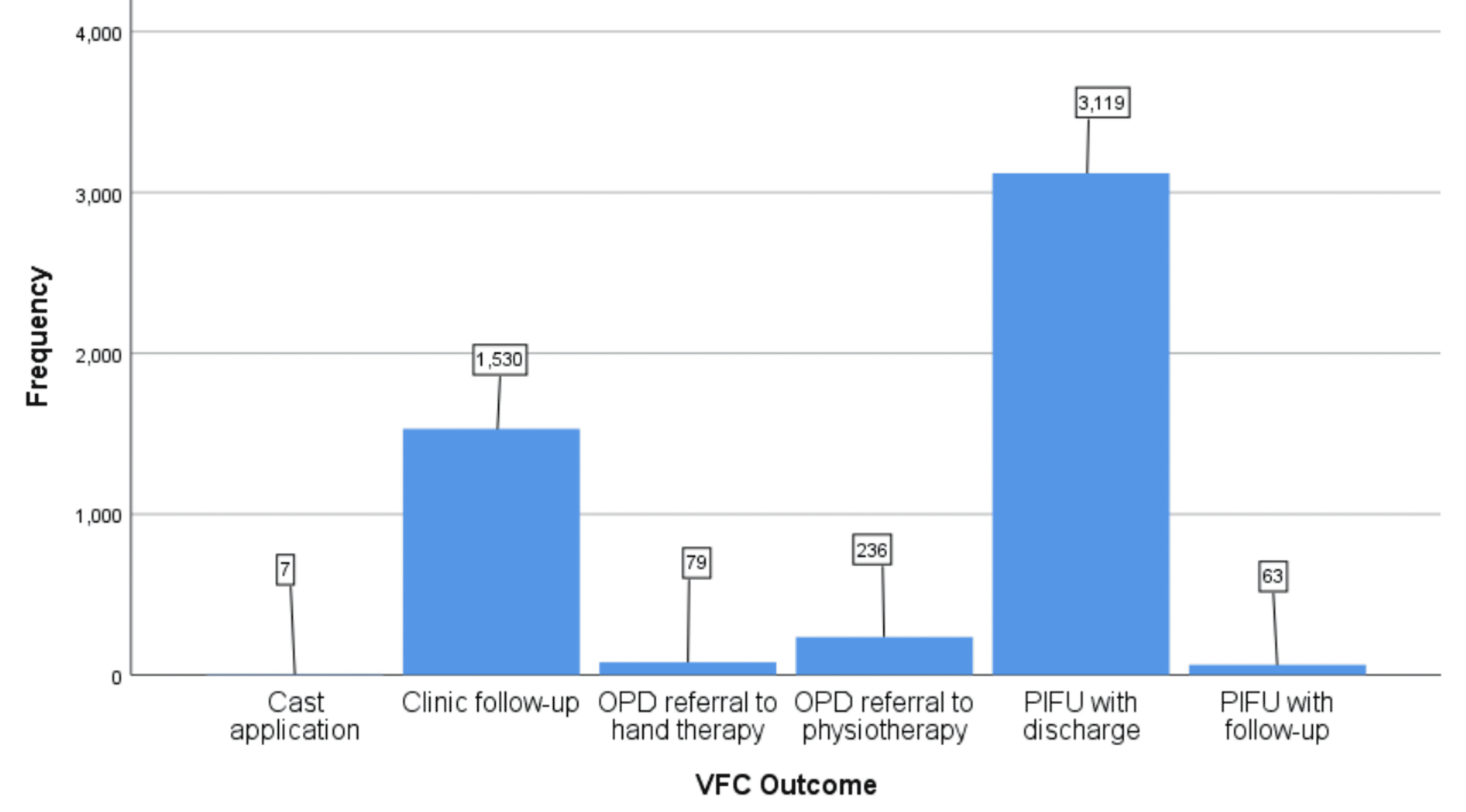
Clinical outcomes of VFC review VFC: Virtual fracture clinic, OPD: Outpatient department; PIFU, Patient-initiated follow-up

Following VFC review, clinical outcomes were documented, including the need for further investigation, face-to-face consultation, referral to physiotherapy or hand therapy, and patient-initiated follow-up (PIFU) with or without discharge. Of the 5,034 patients reviewed, 1,530 (30.4%) required face-to-face consultation, while 3,119 (62%) were discharged with PIFU. Among those discharged with PIFU, 849 (16.9%) were subsequently rebooked into subspecialty fracture clinics for further evaluation. The remaining patients were referred to physiotherapy or hand therapy as indicated (Figure 3). A chi-square test demonstrated a significant association between injury type and VFC outcome (χ² = 1198.9, p <0.001, Φ =0.48), indicating a moderate effect size (Table 1).

**Table 1:** Relationship between injuries and outcome of VFC VFC: Virtual fracture clinic; OPD: Outpatient department; PIFU: Patient-initiated follow-up

Injury	Cast application (n)	Clinic follow-up (n)	OPD referral to hand therapy (n)	OPD referral to physiotherapy (n)	PIFU with discharge (n)	PIFU with follow-up (n)	Total (n)
Arm injury	0	10	1	14	0	0	25
Elbow injury	0	122	0	9	166	6	303
Foot and ankle injury	1	207	1	34	775	17	1035
Forearm injury	0	41	1	0	75	8	125
Foreign body	0	1	0	0	1	0	2
Hand injury	0	153	76	0	747	12	988
Injury of the upper leg	0	0	0	0	0	1	1
Knee injury	1	204	0	102	156	0	463
Lower leg injury	0	41	0	0	35	0	76
Pelvic and hip injury	0	16	0	5	15	1	37
Shoulder injury	0	351	0	78	348	3	780
Wrist injury	5	383	1	7	787	16	1199
Total	7	1530	79	236	3119	63	5034

## Discussion

Both conventional fracture clinics and VFCs adhere to the same core principles: providing high-quality health care, minimising unnecessary hospital visits, reducing excessive medical imaging, and promoting appropriate rehabilitation [10,11]. Several pre-pandemic studies have demonstrated that the VFC model is well-accepted in various local centers for managing specific injuries, including fifth metacarpal fractures [11,12], fifth metatarsal fractures [6,11], clavicle fractures [13], mallet finger injuries [14], and ankle fractures [7]. When formal clinical evaluation is required due to the nature of the injury, a mechanism exists to schedule the patient for the next available subspecialty clinic [15]. A study by Bellringer et al. examining the management of radiologically stable Weber B ankle fractures through VFCs reported a mean cost savings of £237 per patient compared to traditional fracture clinic management [7].

In addition to cost-effectiveness, VFCs have been shown to significantly reduce patient waiting times compared to conventional fracture clinics, as demonstrated in a study by Holgate et al. [16]. Traditional fracture clinics are associated with various indirect costs, including travel expenses, parking fees, time away from work, and environmental impacts from carbon emissions associated with travel, and VFC helps in tackling all these issues [17].

Our study reviewed 5,034 patients referred to the VFC over a 12-month period. No patients were excluded from the sample. Of these, 3,119 patients (62%) were discharged following review by the VFC. However, 849 (16.9%) of these patients were subsequently rebooked into subspeciality fracture clinics for further evaluation, resulting in 2,270 patients (45.1%) not requiring additional follow-up or consultation. These findings align with studies conducted by McKirdy et al. [18] and O'Rielly et al. [19] reporting discharge rates following VFC review ranging from 33% to 60%.

Discharging patients through the VFC reduces the need for face-to-face clinic visits, creating capacity for patients requiring in-person assessments for complex orthopaedic issues. Most patients referred from the emergency department to the VFC presented with upper limb or foot and ankle injuries, which are often manageable nonoperatively through consultant-led VFCs. Patients are provided with specialist advice and safety-netting information during VFC consultations. When further assessment or investigation is required, appropriate investigations are ordered through the VFC, and patients are subsequently reviewed in the next available subspecialty fracture clinic. The VFCs have demonstrated higher patient satisfaction, reduced face-to-face consultations, and safety in delivering care, with most patients receiving specialist input through the VFC [20].

This study has several limitations that should be considered when interpreting the findings. As a retrospective study, the data were extracted solely from hospital record systems, making the study rely on existing records’ accuracy and completeness. The absence of patient feedback is another significant limitation, as this study did not assess patient satisfaction or their perceived quality of care received through the VFC. Additionally, the study did not evaluate clinical outcomes beyond the initial review process, particularly for patients who were discharged with patient-initiated follow-up but later required further assessment. The lack of a control group and the single-centre study design may limit the generalisability of the findings to other institutions or healthcare systems. Finally, potential biases in patient referral patterns or decision-making by clinicians could have influenced the results. Future research should include prospective studies with patient-reported outcomes and comparisons between VFCs and traditional fracture clinics to provide a more comprehensive evaluation of the VFC model.

## Conclusions

This study aimed to evaluate the utilisation of the VFC at Addenbrooke’s Hospital, Cambridge University Hospital NHS Foundation Trust, over a 12-month period and assess its effectiveness in managing orthopaedic patients referred from emergency departments and minor injury units. This study demonstrated that the VFC model effectively manages a substantial proportion of patients referred from emergency departments and minor injury units. Additionally, by creating capacity within conventional clinics, VFCs can enhance access to care for patients requiring in-person assessment and treatment of complex orthopaedic issues. These findings support the continued use and potential expansion of VFCs as a valuable tool in modern orthopaedic care. Future research should include prospective studies incorporating patient feedback to further evaluate the safety, satisfaction, and clinical outcomes associated with VFCs.
